# Tricarboxylic Acid (TCA) Cycle Enzyme Alpha‐Ketoglutarate Dehydrogenase (KGDH) as a Nexus for the Regulation of Macrophage Polarization

**DOI:** 10.1002/jcb.70104

**Published:** 2026-07-05

**Authors:** Meijing Li, Ryan J. Mailloux

**Affiliations:** ^1^ School of Human Nutrition McGill University, Ste Anne de Bellevue Ste Anne de Bellevue Quebec Canada; ^2^ Department of Biochemistry, Faculty of Medicine and Health Sciences McGill University Montreal Quebec Canada

## Abstract

Macrophage metabolism has been increasingly studied in recent years for its potential as a therapeutic target across multiple pathologies. In this article, we propose that the tricarboxylic acid (TCA) cycle enzyme alpha‐ketoglutarate (KG) dehydrogenase (KGDH) serves as a nexus for regulating macrophage polarization toward pro‐inflammatory (M1) or anti‐inflammatory (M2) phenotypes. This is achieved through modulation of mitochondrial hydrogen peroxide (mtH_2_O_2_) and the availability of the TCA cycle metabolites KG and succinate, which are important immunomodulatory molecules. We discuss the evidence showing KGDH is a potent source of mtH_2_O_2_ in various cell types and how it could use this reactive oxygen species (ROS) to modulate signaling pathways involved in macrophage differentiation. Coupled to this, we describe emerging evidence showing that KG and succinate exert opposite signaling effects in macrophages, with the former metabolite inducing an anti‐inflammatory phenotype and the latter (succinate) promoting inflammation. This occurs through the regulation of dioxygenases involved in hypoxic signaling and epigenetic programming and the activation of G‐protein coupled receptor 91 (GPR91) by succinate. Importantly, we contend that regulating KGDH influences the availability of these two metabolites, which, along with controlling mtH_2_O_2_ availability, helps control macrophage polarization. Collectively, increased mtH_2_O_2_ generation and succinate are known to induce a pro‐inflammatory phenotype, whereas low mtH_2_O_2_ and high KG have the opposite effect. This suggests that KGDH is a key regulator of macrophage polarization by controlling the availabilities of these immunomodulatory metabolites.

## 1 Introduction

**FIGURE 1:**
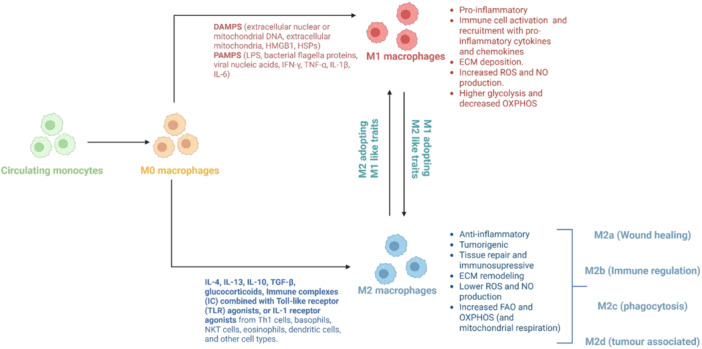
Factors that influence pro‐inflammatory and anti‐inflammatory differentiation. Resident naïve macrophages (M0) or circulating monocytes that differentiate into M_0_ cells can polarize into pro‐inflammatory (M1) or anti‐inflammatory (M2) cells. This is dependent on various factors in the subcellular microenvironment. Pro‐inflammatory differentiation is driven by damage‐associated molecular patterns (DAMPs; endogenous danger signals released by damaged or dying cells) and/or pathogen‐associated molecular patterns (PAMPS; structural or secreted biomolecules from microbes and viruses). DAMPs include extracellular nuclear or mitochondrial DNA, mitochondria, high mobility group box 1 (HMGB1), or heat shock proteins (HSPs). PAMPs are lipopolysaccharide (LPS), viral or bacterial DNA, bacterial flagellar proteins, or pro‐inflammatory cytokines secreted by other M1 macrophages (e.g., IFN‐γ, TNF‐α, IL‐1β, IL‐6). These factors bind to distinct cell‐surface receptors (summarized in Figure 2) to elicit signals that induce the M1 phase. The various genes induced by these pathways to promote inflammation are summarized in the Figure. By contrast, other distinct factors, some of which are secreted by M1 cells, are needed to “turn down” the inflammatory response. These factors promote either the polarization of M0 cells to the M2 phase or the conversion of M1 cells to the M2 phase. These factors include interleukins (IL‐4, IL‐10, IL‐13), TGFβ, glucocorticoids, and immune complexes, which are secreted or presented on the surface of various immune cell types and other cells. The binding of these factors to their receptors induces the M2 phase by activating the gene programs summarized in the Figure. It is important to note that M2 cells can adopt various subtypes depending on the activated signaling pathways and the surrounding physiological environment. These subtypes fulfill different functions. In addition, M2 cells can adopt some M1 traits (and vice versa), which also depends on the cell sub‐environment. This figure was generated with Biorender.

Macrophages are widely distributed in the blood and tissues of the body because these cells are on the front line of immune defense and tissue remodeling, repair, surveillance, and homeostasis [1]. The diverse functions of macrophages mainly depend on their heterogeneity (Figure 1). Once activated, macrophages differentiate into two main subtypes in response to distinct microenvironmental stimuli and signals: pro‐inflammatory (M1) cells and anti‐inflammatory (M2) cells (Figure 1) [2, 3]. The M1/M2 binary classification is useful and simple for distinguishing between pro‐ and anti‐inflammatory macrophages. However, in vivo, these cells have distinct functional subtypes in tissue microenvironments to handle different stages of the immune response. For example, although M1 cells are often characterized as the pro‐inflammatory archetype in contrast to M2, in vivo there is an entire spectrum of “M1‐like” cells with various cellular and metabolic characteristics, some of which even exhibit “M2 cell‐like” traits [4]. However, even though macrophages are highly plastic cells that adopt various differentiated types and subtypes in response to a wide range of stimuli and pathologies, the binary classification system is still useful because M1 and M2 cells have features that distinguish them as either pro‐ or anti‐inflammatory. For example, M2 macrophages are involved in anti‐inflammatory responses, tissue repair, and immunosuppression (Figure 1) [1]. By contrast, M1 macrophages exhibit high microbicidal activity and produce proinflammatory cytokines and chemokines to recruit and activate other immune cells to eradicate pathogens (Figure 1) [5]. The microbicidal response of M1 macrophages requires the activation of reactive oxygen species (ROS) production and nitric oxide (NO) generation, which are used to neutralize pathogens [6]. The main ROS produced in activated M1 cells are superoxide (O_2_^•−^) and H_2_O_2_, which are then used by myeloperoxidase to make the chlorinated and brominated ROS necessary for pathogen destruction [7]. Overall, macrophage M1 or M2 polarization is a precisely regulated process; however, because macrophage polarization depends on a variety of stimuli, altered environments due to certain pathogens and diseases can cause an imbalance between M1 and M2 polarization, resulting in further tissue damage [2, 5]. The prolonged activation of M1 macrophages, for example, causes chronic inflammation, leading to tissue damage and contributing to fibrotic tissue formation [2, 8]. Thus, inhibiting M1 polarization and promoting M2 traits in macrophages may help alleviate inflammation and treat certain diseases.

M1 activation is highly complex and, as described above and below, involves many overlapping signaling pathways that crosstalk with one another, enabling the innate immune system to mount the response needed to eliminate pathogens and/or damaged cells/tissues. Recent work has shown that M1 and M2 polarization also relies on significant rewiring of mitochondrial metabolic pathways, such as the tricarboxylic acid (TCA) cycle, which produces key immunomodulatory metabolites that determine whether macrophages adopt pro‐ or anti‐inflammatory traits [9, 10]. For example, succinate promotes the M1 phase, whereas alpha‐ketoglutarate (KG) induces M2 polarization [11, 12, 13]. Itaconate, which is produced from cis‐aconitate in the TCA cycle, also regulates macrophage polarization [14]. In addition to metabolites in the TCA cycle, complex I of the electron transport chain (ETC) has been shown to induce M1 polarization by overproducing O_2_^•−^ via reverse electron transfer (RET) from succinate [15]. Similarly, O_2_^•−^ from complex III of the ETC has also been found to drive the M1 phase [16]. The O_2_^•−^‐ induced M1 polarization first requires its conversion to H_2_O_2_, which then induces or prolongs pro‐inflammatory cascades through site‐specific cysteine oxidations in proteins [17]. Therefore, mtH_2_O_2_ also contributes to macrophage polarization. Importantly, mitochondria can contain up to 16 O_2_^•−^/H_2_O_2_ sources, which means generators other than complexes I and III could also promote M1 traits in macrophages. Our research team recently identified KGDH as a potent mtH_2_O_2_ generator in various cell types. Thus, it is feasible KGDH may also supply the mtH_2_O_2_ necessary to promote the pro‐inflammatory phase. Coupled with the observation that KG and succinate serve as immunomodulatory metabolites for macrophage polarization, we propose here that KGDH is a regulatory nexus for the adoption of M1 or M2 traits in macrophages. We posit that temporary inhibition of KGDH is crucial for promoting the M2 phase because it would increase the KG/succinate ratio and abrogate mtH_2_O_2_ production. By contrast, activating KGDH has the opposite effect, decreasing this ratio and promoting mtH_2_O_2_ production, resulting in M1 traits. In addition, the activity of KGDH produces succinyl‐CoA, which has also been implicated in M1 polarization through the succinylation of protein lysine residues. In the sections below, we discuss this new regulatory function of KGDH in the contexts of inflammatory signaling, mtH_2_O_2_‐activated redox communication pathways, and the evidence demonstrating that succinate and KG, along with succinyl‐CoA, are crucial immunomodulatory metabolites. We also highlight this alongside recent evidence from our group showing that KGDH is a highly potent source of mtH_2_O_2_ that outpaces complexes I and III. In aggregate, we propose that targeted, temporary disruption of KGDH activity could be used to treat inflammatory diseases.

## 2 **H**_2_ **O**_2_ is Needed for M1 Polarization

### 2.1 How Does H_2_O_2_ Orchestrate Macrophage Polarization?

Reactive oxygen species (ROS) is an umbrella term that describes all oxyradicals formed by abiotic and biotic systems. The proximal ROS formed in mammalian cells are O_2_^•‐^ and H_2_O_2_, which, if allowed to accumulate, can produce the hydroxyl radical (OH^•^) via the Fenton and Haber‐Weiss reactions [18]. It is OH^•^ that induces most of the damage associated with oxidative distress because it is highly reactive towards biological macromolecules (e.g., proteins, DNA, membranes) and other cell constituents [19]. H_2_O_2_ has attracted the most attention from researchers in the life sciences over the past several decades because it causes oxidative distress and cell death at high levels (e.g., > 100 nM) but also induces cell signals required for proliferation, development, differentiation, and stress adaptation when its levels are low (1‐100 nM; called oxidative eustress) [20]. It is generally accepted that H_2_O_2_ is a second messenger that is crucial for embryogenesis, tissue development, cell migration, neural activity, and wound healing [21, 22]. Low‐dose H_2_O_2_ exposure promotes M1 polarization in both zebrafish and N9 microglial cells [23]. Postpartum hemorrhage also revealed that H_2_O_2_ promotes the polarization of resident macrophages in the placenta towards the M1 phase, thereby triggering inflammatory cell infiltration [24].

**FIGURE 2:**
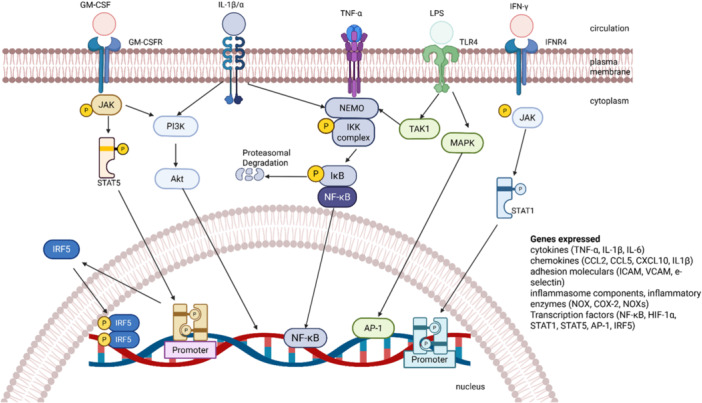
A summary of the major signaling pathways involved in M1 polarization. PAMPS, LPS and cytokines, including GM‐CSF, TNF‐α, IL1‐α/β, and IFN‐γ, binding to their cognate receptors (LPS binds toll‐like receptor; TLR, GM‐CSF binds GM‐CSF receptor; GM‐CSFR, TNF‐α binds TNF receptor, TNFR; IL1‐α/β binds to IL1 receptor, IL1R; IFN‐γ binds IFN‐γ receptor; IFNGR) stimulate phosphorylation cascades that involve: (1) recruitment and phosphorylation of JAK or IκB kinase (IKK) via recruiting NEMO directly or through TAK1 and (2) binding and phosphorylation of STAT1, STAT5, or IκB. In the STAT pathways, phospho‐STAT1 or phospho‐STAT5 form a phosphorylated homodimer, activating its transcriptional activity. Receptor binding also leads to (3) activation of the PI3K Akt pathway or (4) activation of the MAPK and AP‐1. STAT homodimers induce distinct gene transcription programs critical for M1 polarization. In the case of STAT5, the pathway induces the expression of the gene encoding interferon regulatory factor 5 (IRF5), which, upon phosphorylation, also forms a homodimer that activates pro‐inflammatory gene transcription programs. Finally, LPS activates TLR, leading to the induction of IKK and the subsequent phosphorylation and degradation of IκB. This results in NF‐κB migration into the nucleus and the transcription of pro‐inflammatory genes. This figure was generated with Biorender.

H_2_O_2_ promotes macrophage polarization towards the M1 phase by serving as an overlapping signal with the canonical pro‐inflammatory communication pathways described in Figure 2 [25]. H_2_O_2_ is a good second messenger because (1) in comparison to other ROS, it is relatively stable and therefore has a longer half‐life [26], (2) it can be a good electrophile due to the polarizability of the O‐O bond [26], and (3) can cross biological membranes with the aid of aquaporins (AQP) [27]. All these properties make H_2_O_2_ an ideal transmitter of redox signals and regulator of cell functions. H_2_O_2_ propagates oxidative eustress signals through the reversible oxidation of protein cysteine thiols [20, 28]. For this to occur, the cysteine sulfur must first ionize to form a nucleophilic thiolate anion that then attacks the O‐O bond in H_2_O_2_ [26]. This reaction forms a sulfenic acid (SOH), which changes the conformation and/or of a target protein, thereby altering the downstream signaling pathways [15, 29]. Note that SOH formation is not the only redox modification that occurs on protein cysteine thiols. There are many others, including S‐nitrosylation, S‐glutathionylation, S‐acylation, persulfidation, and more, which have been reviewed elsewhere [30].

An overview of major signaling pathways used in M1 polarization is found in Figure 2. These pathways have been described in detail in other excellent reviews [31, 32]. NF‐кB is a well‐known master activator of inflammation and M1 polarization. In M0 phase (e.g., when the cells are not induced to become pro‐inflammatory), NF‐κB is retained in the cytoplasm of the naïve macrophages by inhibitor of nuclear factor kappa B (IκB). Once the pathway is activated by factors such as lipopolysaccharide (LPS), tumor necrosis factor‐alpha (TNF‐α), and interleukin‐1‐beta/alpha (IL‐1β/α), IκB is phosphorylated by IκB kinase (IKK), which targets IκB for proteasomal degradation [32, 33]. This results in NF‐κB migration into the nucleus, where it activates the pro‐inflammatory gene programs required for M1 polarization (Figure 2) [33, 34, 35, 36, 37]. The IKK pathway is dephosphorylated and deactivated by phosphatase and tensin homolog (PTEN), which is vital for controlling the extent of the pro‐inflammatory response [38, 39]. Notably, PTEN activity depends on a catalytic cysteine residue required for IKK dephosphorylation. The active site cysteine in PTEN is a target for H_2_O_2_‐mediated oxidation to SOH [40]. This deactivates PTEN, prolonging NF‐κB activation. In addition, H_2_O_2_‐mediated deactivation of PTEN prolongs activation of other signaling cascades that crosstalk with NF‐κB, such as phosphatidylinositol 3‐kinase (PI3K) and protein kinase B (PKB, a.k.a. AKT), a key pathway required for cell growth and proliferation [41]. The PI3K‐PKB pathway also triggers mTORC1/2 and induces glycolytic and anabolic programs, thereby reinforcing M1 polarization pathways [42]. The H_2_O_2_‐mediated inhibition of phosphatases that desensitize inflammatory signals also promotes induction of the mitogen‐activated protein kinases (MAPKs) pathway, which is composed of p38, extracellular signal‐regulated kinases (ERK), and the c‐Jun N‐terminal kinases (JNKs) [43]. This is achieved by H_2_O_2_−mediated inhibition of dual‐specificity phosphatases (DUSPs) and general protein serine/threonine phosphatases (such as PP2A) by SOH formation in the active site of the enzymes [44, 45]. H_2_O_2_−mediated activation of the MAPK pathway serves as an overlapping signal that reinforces NF‐κB and induces cell proliferation, growth, and survival. Finally, STAT1 in the JAK/STAT pathway also promotes M1 polarization and has been shown to exhibit prolonged activation in response to H_2_O_2_ due to inhibition of protein tyrosine phosphatases (PTPs) [46, 47]. In sum, H_2_O_2_ reinforces pro‐inflammatory signals by inhibiting the phosphatases required to desensitize several phosphorylation/kinase signaling cascades. However, many questions remain regarding the other targets H_2_O_2_ may act on in naïve macrophages and how these H_2_O_2_ redox signals promote M1 polarization.

As described above, H_2_O_2_ is well known to promote inflammation. However, other studies have also shown that H_2_O_2_ can, in certain contexts, promote M2 polarization without playing any role in inflammatory signaling. For example, Zhang et al. used the ROS scavengers butylated hydroxyanisole (BHA), TEMPO, and NAC; the NOX inhibitor apocynin; and NOX knockout to show that ROS is essential for M2, not M1, activation [48]. It is important to note that this difference in the immunomodulatory role of H_2_O_2_ could be due to the microenvironment in which the population of macrophages being studied was. In Zhang et al., the authors found that H_2_O_2_ promoted immunosuppression through M2 induction in a tumor microenvironment. In this case, tumor‐associated macrophages (TAMs) use the excess H_2_O_2_ produced by neighboring cancer cells to induce M2 traits in naïve macrophages while suppressing M1 polarization, thereby promoting tumor metastasis [48]. Currently, it is not well understood how the tumor microenvironment promotes M2, rather than M1, traits in response to H_2_O_2_. However, it does demonstrate the complexities of how cells transmit and propagate intra‐ and intercellular redox signals and how this may affect subpopulations of cells, such as macrophages. Recent articles have proposed that H_2_O_2_−mediated redox signals serve as an important interface that connects the exposome, which is the measure of all the environmental exposures an individual cell encounters over its lifetime, and the regulation of the genome, epigenome, transcriptome, proteome, and metabolome [17, 49]. The role of H_2_O_2_ in fulfilling such a role is complicated even further by the fact that individual proteins can have one or more oxidizable cysteines and therefore adopt distinct oxidation forms or “oxiforms” [30]. As discussed above in these reviews, proteins can also undergo many other types of reversible sulfur oxidations, which can also have profound effects on cell adaptations and cell fate decisions. Advances in molecular biology tools that detect H_2_O_2_ and other ROS have significantly increased our understanding of how redox signals in cells are propagated and how these signals are dependent on spatio‐temporal changes in H_2_O_2_ levels in cellular microdomains and sub compartments. But many questions remain to be answered regarding how H_2_O_2_ signals are transmitted in cells and the role these H_2_O_2_ signals play in physiological and pathological outcomes.

### 2.2 A General Overview of H_2_O_2_ Sources and Sinks in Mammalian Cells

Over 60 ROS generators have been identified in mammalian cells, and these producers form only O_2_^•−^ or a mixture of O_2_^•−^/H_2_O_2_ in the extracellular milieu, the cytoplasm, or in various organelles (e.g., mitochondria, the endoplasmic reticulum (ER), peroxisomes, nucleus) [49]. Of these sources of O_2_^•−^ and/or H_2_O_2_, 16 are located in mitochondria [49, 50]. Mitochondrial flavin‐dependent enzymes produce both O_2_^•−^ and H_2_O_2_ and non‐flavin enzymes generate only O_2_^•−^ [51]. If O_2_^•−^ is produced, then it is rapidly dismutated to H_2_O_2_ by superoxide dismutase (SOD) [52, 53]. Cells express three SOD isozymes: SOD1 (cytoplasm and mitochondrial intermembrane space (IMS)), SOD2 (mitochondrial matrix), and SOD3 (extracellular space) [54]. Main cellular O_2_^•−^ and/or H_2_O_2_ sources include nicotinamide adenine dinucleotides phosphate (NADPH) oxidases (NOXs; plasma membrane, cytoplasm, and endoplasmic reticulum), the tricarboxylic acid (TCA) cycle enzymes pyruvate dehydrogenase (PDH) and KGDH and electron transport chain (ETC) complexes I, II and III in mitochondria, and endoplasmic reticulum (ER) oxidoreductin‐1 (ERO1) and cytochrome P 450‐monooxygenases [53, 55]. Any O_2_^•−^ formed in these compartments or in the extracellular environment is rapidly converted by SOD1‐3 to H_2_O_2_, which is then degraded by the glutathione (GSH, thioredoxin (TRX), and catalase systems [56]. However, as described above, H_2_O_2_ is used to propagate oxidative eustress signals. H_2_O_2_ also lasts longer in cells when compared to O_2_^•−^ because it is not neutralized as fast. O_2_^•−^ has a very short half‐life in cells and the extracellular environment because SOD dismutates it to H_2_O_2_ at 10^9 ^M^−1^ s^−1^ [53]. O_2_^•−^ also cannot cross biological membranes because its negative charge is repulsed by the phospholipid bilayer [57]. By contrast, H_2_O_2_ removal occurs at 10^5^−10^7 ^M^−1^ s^−1^, which allows it to persist longer in cell environments and cross biological membranes using AQP [27]. These factors make H_2_O_2_ the ideal ROS for transmitting oxidative eustress signals.

### 2.3 NADPH Oxidase (NOX) Is a Crucial H_2_O_2_ Source in Macrophages

Apart from mitochondria, NOX is considered to be a major source of H_2_O_2_ in macrophages. The NOX family consists of NOX1, NOX2, NOX3, NOX4, NOX5, DUOX 1 and 2 [58]. NOXs generate O_2_^•−^ whereas the DUOXs produce H_2_O_2_ because of a unique peroxidase domain in the enzyme [59]. NOXs have two main domains required for their catalytic function: the cytosolic dehydrogenase domain and the heme‐coordinating transmembrane domain. The DH domain comprises two lobes that form the FAD and NADPH binding sites, respectively [60]. Upon activation, NADPH is bound and oxidized, resulting in the reduction of FAD to FADH_2_, which is then oxidized for the transfer of two electrons to molecular oxygen (O_2_) through the proximal and distal heme of the cytochrome in NOX, resulting in O_2_^•−^ production [61]. Based on the location and current evidence, three of these NOXs are associated with macrophage polarization: NOX1 expressed on the plasma membrane, NOX2 expressed on the plasma membrane and phagosome membrane, and NOX4 located in the intracellular membrane, including ER and mitochondria [62, 63]. This illustrates that the NOX family of enzymes can produce O_2_^•−^/H_2_O_2_ in different cellular subcompartments, thereby fulfilling its many signaling functions. The NOXs are viewed as being essential for M1 polarization, but a few studies have challenged this concept. For example, M1 polarization was observed in cells attenuated for NOX1 and NOX2, suggesting that other ROS generators are involved in the induction of inflammation [64, 65]. This contrasts with other studies showing that the genetic or pharmacological disruption of NOX1 and NOX2 mitigates inflammatory cytokine production [66, 67, 68, 69]. Surprisingly, the role of NOX4 in inflammation has also shown inconsistent results across studies, with some data indicating it contributes to M1, while others suggest it promotes the M2 phase [63, 67, 70]. Therefore, although the NOX family is crucial for macrophage function, other sources of H_2_O_2_, especially those produced from mitochondria, also require more studies to confirm their role in polarization.

### 2.4 M1 Macrophage Polarization Depends on Mitochondrial H_2_O_2_ Production

As described above, there are 16 estimated sites of O_2_^•−^ and/or H_2_O_2_ production in mitochondria. Among these sites, complexes I and III of the ETC are historically viewed as the primary sources of O_2_^•−^ and/or H_2_O_2_ [71, 72, 73]. Complex I is an NADH ubiquinone oxidoreductase that is comprised of 3 modules (the N‐module, Q‐module, and P‐module) [74]. The N‐module makes contact with the matrix and contains the NADH binding site and flavin mononucleotide (FMN) and 8 Fe‐S clusters that conduct electrons from NADH to coenzyme Q_10_ in the binding pocket of the Q‐module [75, 76]. NADH is produced by the TCA cycle, and the pathways that converge on the TCA cycle (e.g., branched‐chain amino acid metabolism) are oxidized by the N‐module. This injects 2 electrons into complex I, which are then transferred to coenzyme Q10 (CoQ) [75, 76, 77, 78]. Complex II (succinate dehydrogenase; SDH) is composed of four subunits and transfers electrons to CoQ from the TCA cycle metabolite succinate through an FAD prosthetic group. Reduction of CoQ by complexes I and II forms CoQH_2_, which is then oxidized by complex III. Complex III then passes the electrons through the Rieske Fe‐S cluster subunit (ISP) and cytochrome c_1_ to cytochrome c [79, 80]. The electrons are then used to reduce O_2_ to H_2_O at complex IV. Oxidation of NADH and the subsequent conductance of electrons through this chain results in the pumping of 10 protons into the intermembrane space: 4H^+^ by complex I, 2H^+^ by complex III, and 4H^+^ by complex IV. This establishes the protonmotive force (pmf) necessary to drive ATP synthesis by complex V. Succinate oxidation only pumps 6H^+^ into the IMS because it bypasses complex I, and SDH is not involved in the formation of the pmf. However, as described below, succinate oxidation can result in reverse electron transfer (RET) to complex I, which can be a major source of mtH_2_O_2_ [81, 82]. Electron flow through the ETC is not perfectly coupled to the oxidative phosphorylation (OxPhos) of ADP. At certain points in the chain, these electrons can prematurely "spin‐off" and react with O_2_ forming ROS.

Complexes I and III of the ETC are defined as the main ROS generators in mitochondria. However, recent work has challenged the notion that complex I and III are the main O_2_^•−^ and/or H_2_O_2_ sources in mitochondria by showing the other “unconventional” mitochondrial generators, like KGDH, can outpace the ETC in mtH_2_O_2_ production. It is important to note that although it is possible the overall contributions of complex I and complex III to total H_2_O_2_ production may have been overestimated, it does not minimize their contributions to mtH_2_O_2_ signaling in macrophage differentiation [83]. For example, a recent study by Casey et al. showed that complex I‐mediated mtH_2_O_2_ production following LPS stimulation of macrophages is necessary for M1 polarization [15]. In the study, Casey et al. found succinate was the main driver of this mtH_2_O_2_ production, which occurred by reverse electron transfer (RET) from complex II of the ETC to complex I [15]. This mtH_2_O_2_ is then used to assemble inflammasomes. Complex I inhibition decreased TNF‐α, IL‐6, and IL‐1β, demonstrating that succinate‐driven mtH_2_O_2_ genesis by RET is needed for pro‐inflammatory signaling [15]. In addition, recent studies have shown that complex III‐generated O_2_^•−^ is a specific redox signal essential for IL‐10 induction, which is necessary for M2 polarization [84].

It is important to underscore that complexes I and III are not the only sources of H_2_O_2_ in pro‐inflammatory signaling. In our view, other important ROS generators, such as KGDH, could also contribute to M1 polarization. Our team has found that KGDH is a potent H_2_O_2_ producer in mouse liver mitochondria and several cell lines [83, 85]. KGDH accounts for the majority of the mtH_2_O_2_ production in mouse liver mitochondria, the Huh‐7 hepatocellular carcinoma, and HepG2 hepatoblastoma cell lines metabolizing various fuels (e.g., glucose, pyruvate, lactate, amino acids or fatty acids) [83, 85]. PDH and dihydroorotate dehydrogenase (DHODH) were also shown to be important mtH_2_O_2_ generators in these model systems [83, 85]. Surprisingly, in these same experiments, complexes I and III accounted for a limited amount of the total H_2_O_2_. As described below, KGDH was recently identified as a key regulator of M1 and M2 polarization by controlling the availability of KG and succinate. Notably, other mtH_2_O_2_ sources, such as PDH and DHODH, should be considered, as both have been identified as important H_2_O_2_ generators. When taken together, it is clear that mtH_2_O_2_ is vital for M1 polarization, and the sources of this mtH_2_O_2_ can be the ETC and potentially KGDH.

## 3 Macrophage Polarization Depends on Metabolic Reconfiguration

**FIGURE 3:**
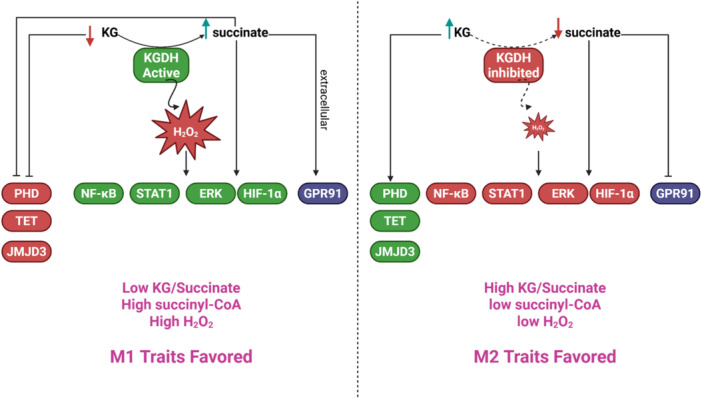
Proposed mechanism for the regulatory function of KGDH in macrophage differentiation. *Left*: Increased KGDH activity augments mtH_2_O_2_ production and decreases the KG/succinate ratio, promoting M1 traits. This is achieved through the H_2_O_2_ mediated inhibition of phosphatases that desensitize NF‐κB, MAPK, and STAT1. Succinate stimulates GPR91 and inhibits KG‐dependent dioxygenases like PHD, JMJD3, and TETs. *Right*: The inhibition of KGDH promotes the anti‐inflammatory phase by abrogating succinate and succinyl‐CoA production and limiting mtH_2_O_2_ genesis. Diminished succinate availability limits GPR91 signaling and the lowered rate of mtH_2_O_2_ genesis preserves phosphatase activity. KG also builds up, which is a key cofactor for several dioxygenases that regulate macrophage polarization. This figure was generated with Biorender.

The concept that KGDH serves as a regulatory nexus for macrophage differentiation is summarized in Figure 3 and discussed in more detail in Section 6 below. The reconfiguration of metabolite flux through the TCA cycle is crucial for mammalian cell growth, proliferation, differentiation, and the induction of cell stress coping strategies. For example, it has been known for some time that the TCA cycle is rewired in cells experiencing low molecular oxygen (O_2_) to promote succinate buildup for the activation of the hypoxic response [86]. TCA cycle was also found to be reconfigured in response to oxidative distress to promote antioxidant defense through the increased biosynthesis of NADPH and the accumulation of KG, which spontaneously scavenges H_2_O_2_ producing succinate [87]. Cancer cells also reconfigure the TCA cycle in many ways to sustain rapid cellular growth, promote survival, and induce metastasis [13, 88]. However, the concept the retailoring of the TCA cycle in the regulation of macrophage polarization is a relatively new one. Both M1 and M2 cells depend on TCA cycle reconfiguration to produce the immunometabolites necessary that promote pro‐ or anti‐inflammation phenotypes [8, 89]. Oxidative phosphorylation (OxPhos) is the primary metabolic pathway for ATP production in anti‐inflammatory macrophages, while the presence of pro‐inflammatory stimulants promotes glycolysis to satisfy energy demands in M1 cells [90, 91]. Glycolysis is a key player in the inflammatory response because it supplies the intermediates and ATP necessary for cell proliferation in environments with limiting O_2_ levels (e.g., when macrophages need to clear an infection) [92]. Under physiological conditions, M1 cells use glycolysis to meet increased energy demands in hypoxic tissue environments at sites of inflammation, where the O_2_ saturation is very low [92]. This upregulation of glycolysis also supplies glucose‐6‐phosphate for the pentose phosphate pathway, which produces NADPH, the substrate needed for O_2_^•−^ and NO production by NOX and iNOS, respectively [93, 94, 95]. LPS and IFN‐γ induce the metabolic switch from OxPhos to glycolysis, and the interruption and inhibition of glycolysis impairs M1 macrophage function and the inflammatory response [96, 97]. Studies have also reported that the inhibition of glycolysis can suppress M1 polarization and shift it toward the M2 phenotype [98, 99]. M2 macrophages use fatty acid oxidation (FAO) and OxPhos instead of glycolysis as a main source of ATP [100]. By contrast, current research provides limited evidence that increased FAO promotes M2 polarization, which is also associated with increased OxPhos [101, 102]. In general, these findings demonstrate that macrophage polarization involves significant metabolic rearrangements to meet ATP demands in response to the availability of fuels and substrates and to supply the metabolites necessary for proliferation, growth, and signaling. Most notably, the TCA cycle is at the center of these reconfigurations. It is rewired to produce key immunometabolites, such as KG, succinate, succinyl‐CoA, and others (e.g., itaconate), to orchestrate M1 and M2 polarization.

## 4 Succinate Is an Immunometabolite That Promotes M1 Polarization

**FIGURE 4:**
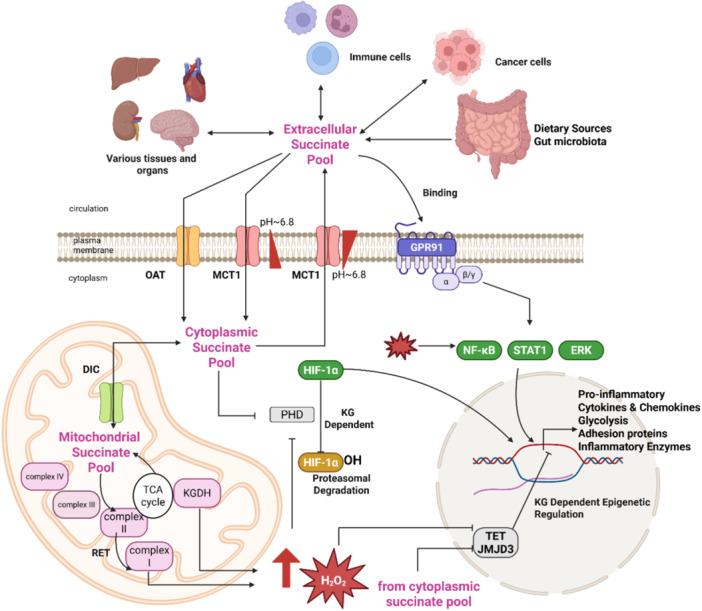
Succinate is a key immunomodulatory metabolite that promotes the pro‐inflammatory phase. Succinate can be produced by endogenous metabolism in macrophages or supplied from various exogenous sources including the diet and gut microbiome, various tissues (e.g., heart, muscle, brain, kidneys), other immune cells, and by tumor cells. The extracellular succinate pool is highly dynamic, as it is influenced by multiple physiological and pathological processes. Succinate import is facilitated by organic anion transporter (OAT) or monocarboxylate transporter‐1 (MCT1). Note that succinate uptake by MCT1 is dependent on pH (e.g., the monocarboxylate form of succinate is imported) and that MCT1 is reversible. Exogenous succinate also binds to G protein‐coupled receptor 91 (GPR91), activating signaling programs that operate in tandem with the pathways summarized in Figure 2. This reinforces pro‐inflammatory signaling cascades to promote M1 differentiation. The cytoplasmic succinate pool can be used to directly inhibit prolyl hydroxylase (PHD), resulting in hypoxia inducible factor‐1α (HIF‐1α) stabilization and the activation of glycolytic genes. Succinate can also migrate to the nucleus and interfere with α‐ketoglutarate (KG)‐dependent dioxygenases involved in DNA and histone demethylation. Succinate also drives high rates of H_2_O_2_ production by mitochondria. This requires entry into the matrix through dicarboxylate carrier (DIC) and its metabolism by complex II. Succinate also exits mitochondria using the DIC. Reverse electron transfer (RET) to complex I promotes mtH_2_O_2_ production but the further metabolism of fumarate and other metabolites through the TCA cycle can also promote mtH_2_O_2_ genesis by α‐ketoglutarate dehydrogenase (KGDH). Note that KGDH activity regenerates succinate which can also be used in intra‐ and intercellular signaling.

Physiological succinate homeostasis is highly complex because its availability is influenced by cellular metabolism, dietary patterns, gut microbiota composition, and physiological and pathological factors (Figure 4). Uptake and efflux of succinate occur through the pH‐gated monocarboxylate transporter‐1 (MCT1) or organic anion transporter (OAT), which, upon entry into macrophages, is used in signaling and metabolism (Figure 4). Succinate is well documented as an intra‐ and intercellular signaling molecule that regulates M1 polarization. For example, a recent study using succinate‐loaded tumor cell‐derived microparticles to deliver succinate to tumor‐related macrophages successfully promoted classical M1‐like polarization [103]. In addition, succinate has already been identified as a pro‐inflammatory marker in the early stages of metabolic dysfunction‐associated steatotic liver disease (MASLD), a disease that advances to more serious forms due to chronic inflammation. This further supports the idea that succinate is used as a proinflammatory factor [104].

### 4.1 Control of Succinate Availability by Redox Signals and Succinate Accumulation

Tissue hypoxia and inflammation complement each other, and within the hypoxic environment, studies have demonstrated that succinate acts as a signaling molecule [105]. In fact, the role of succinate in hypoxic signaling was first described in pheochromocytomas, gliomas, and paragangliomas, which accumulate due to mutations in the gene(s) encoding complex II subunits *Sdha*, *Sdhb*, *Sdhc*, or *Sdhd* [106]. Under healthy conditions, succinate is efficiently oxidized by SDH (described above). When the environment is hypoxic, SDH reverses its activity, converting fumarate into succinate, which contributes to succinate accumulation [107]. In addition, other pathways for succinate production, including glutamine‐dependent cataplerosis to α‐ketoglutarate and the gamma‐aminobutyric acid (GABA) pathway (Figure 4) [108]. The accumulated succinate is then transported into the cytosol via dicarboxylate carriers (DIC; SLC25A10) and voltage‐dependent anion channels (Figure 4) [109, 110]; followed by the succinate efflux into the extracellular environment by sodium (Na^+^)/succinate symporter (SLC13A3/NaDC3), pH‐gated succinate carriers (e.g., MCT1) (Figure 4) [111]. As mentioned before, metabolic rewiring during M1 polarization results in elevated glycolytic activity and reduced oxidative phosphorylation. Metabolomic analysis of M1 macrophages revealed that the disruption of IDH and SDH causes a discontinuity in TCA cycle flux, resulting in the buildup of citrate and succinate [112, 113, 114]. Itaconate is a potent SDH inhibitor and therefore a key contributor to macrophage immunoregulation [14]. Disrupting itaconate production has shown that the absence of itaconate leads to abrogation of succinate accumulation. Meanwhile, it competitively binds to SDH, making it a crucial player in studying succinate's contribution to M1 polarization [114].

### 4.2 *S*uccinate Accumulation Enhances mtH_2_O_2_ Generation

Succinate is a four‐carbon intermediate in the TCA cycle, and it is produced from the cleavage of the high‐energy thioester bond in succinyl‐CoA by succinyl‐CoA synthetase [115]. Succinate is then oxidized to fumarate by SDH, which, as described above, injects electrons into the CoQ pool of the ETC [116]. SDH serves a dual role in the TCA cycle and the ETC because it couples TCA cycle flux to the two‐electron reduction of the CoQ‐pool, giving succinate a unique role in both central mitochondrial metabolism and aerobic respiration [116]. Under certain (patho)physiological conditions, succinate can accumulate in mitochondria, sometimes to low millimolar levels, causing it to spill into the cytoplasm and extracellular matrix. Succinate is exported by mitochondria into the cytoplasm by the transporters described above [117] (Figure 4). When succinate accumulates in mitochondria, its oxidation by complex II can drive RET to complex I, which can cause excessive O_2_^•−^ production (Figure 4) [116]. As discussed above, this O_2_^•−^ hyper‐generation increases H_2_O_2_ availability, which then acts as a signaling mechanism linking succinate buildup to the activation of pro‐inflammatory responses. The increased H_2_O_2_ levels prolong NF‐κB, STAT1, STAT5, and ERK signaling by inhibiting phosphatases that desensitize these pro‐inflammatory pathways (Figure 4). This occurs through the oxidation of catalytic sulfurs in the phosphatases to sulfenic acids. H_2_O_2_ also promotes M1 traits through the inhibition of prolyl hydroxylases (PHD), which leads to the stabilization of HIF‐1α (described in more detail below) (Figure 4). In addition, H_2_O_2_ promotes inflammasome assembly and could also inhibit JMJD3 and TET enzymes, which are involved in modulating the epigenome (Figure 4). In sum, succinate promotes M1 polarization by enhancing mtH_2_O_2_ generation, a crucial second messenger in pro‐inflammatory signaling that activates multiple pathways promoting M1 traits.

### 4.3 Succinate Inhibits Dioxygenases, Which Promotes Hypoxic Signaling and Epigenetic Regulation in Macrophages

KGDH activity produces succinate, which can promote hypoxia signaling pathways and influence epigenetic regulation by inhibiting dioxygenases. The transcription factor hypoxia‐inducible factor‐1 (HIF‐1) is a heterodimer composed of HIF‐1α and HIF‐1β that plays an essential role in M1 polarization [118]. HIF‐1 is activated by cell hypoxia, specifically through the stabilization of HIF‐1α (Figure 4) [118]. This is a crucial cellular response to O_2_ availability because HIF‐1 activation induces the expression of glycolytic genes to help maintain cellular ATP levels [118]. As mentioned in the first section, enhanced glycolysis is a distinct characteristic of M1 macrophages, which is promoted by HIF‐1α stabilization. Attenuation of HIF‐1α in mice prevents the induction of the inflammatory response, demonstrating HIF‐1 activation is essential for M1 polarization [119]. HIF1‐α is degraded rapidly under normoxic conditions by the proteasome after its hydroxylation by prolyl hydroxylase (PHD1‐3) and ubiquitination by the von Hippel‐Lindau (VHL) protein/E3 ubiquitin ligase complex [120]. PHDs are KG‐dependent dioxygenases (2‐oxoglutarate‐dependent dioxygenases; 2OGDD), and their activity depends on KG, Fe, and ascorbate as cofactors. Succinate is the product resulting from the binding of KG to the 2OGDD for its function [121], which means a higher level of succinate in comparison to KG will slow down 2OGDD's activity. Thus, succinate accumulates during hypoxia and indirectly stabilizes HIF1‐α levels by inhibiting PHD2 activity [122]. In addition, overproduction of mtH_2_O_2_ by complexes I and III due to succinate buildup also stabilizes HIF‐1α by inhibiting PHD (Figure 4) [123]. Although it is established that ROS from the ETC stabilizes HIF‐1α, Gobelli et al. recently showed that excessive H_2_O_2_ genesis induced by attenuating Sdha and Sdhb expression in RAW264.7 macrophages impedes HIF‐1α signaling, but does not interfere with pro‐inflammatory signaling [124]. Other 2OGDD enzymes, such as ten‐eleven translocation (TET) and JMJD3 (Jumonji domain‐containing protein‐3, also known as KDM6B), which are KG‐dependent dioxygenases critical for epigenetic regulation, are also inhibited by succinate (Figure 4) [125, 126]. TET oxidizes 5‐methylcytosine (5mC) in DNA, converting it to (5)‐hydroxymethylcytosine (5hmC), and JMJD3 is a histone demethylase. JMJD3 was recently found to play a key role in M2 polarization by catalyzing the removal of trimethyl groups from histone 3 lysine 27 (H3K27me3) [127]. The JMJD3‐mediated M2 polarization is blocked by experimentally increasing succinate levels [126]. In contrast to JMJD3, the role of succinate in modulating the inflammatory epigenetic landscape through TET enzyme regulation is more complex. Indeed, TET enzyme activation has been shown to resolve inflammation, such as through the activation of regulatory T‐cells, but in other cases TETs can promote inflammation in response to viral infections or certain cancers [125, 128, 129]. There are three TET isoforms (TET1, TET2, TET3), each encoding multiple variants generated by alternative promoter usage or splicing [130], and a more detailed discussion of the TET enzyme in combination with KG is in Section 5. Overarchingly, all isoforms use KG to catalyze the conversion of 5mC to 5hmC and are thus likely to be tightly modulated by succinate inhibition (Figure 4). Itaconate, another potent immunosuppressive metabolite produced by the TCA cycle, was recently identified as an inhibitor of TET, which dampens the pro‐inflammatory response [131]. When taken together, succinate produced by KGDH activity likely plays an integral role in regulating the pro‐inflammatory response by inhibiting 2OGDDs that control hypoxic signals and epigenetic coding.

### 4.4 Succinate Promotes M1 Polarization Through GPR91

Succinate is recognized as a paracrine and endocrine signaling molecule that induces changes in cell function(s) by binding G protein–coupled receptor 91 (GPR91, a.k.a., succinate receptor‐1 [SUCNR1]) (Figure 4). GPR91 belongs to the family of G protein‐coupled receptors (GPCRs), seven‐transmembrane‐domain proteins that bind to a diverse range of extracellular ligands and mediate intracellular signaling cascades by coupling to G proteins and arrestins [132]. There are over 800 *Gpcr* genes in the human genome that are further diversified by alternative splicing and other mechanisms, resulting in hundreds more isoforms [133]. Although some of these GPCRs are still classified as orphan G‐proteins, they are considered major targets for a multitude of therapeutics, which account for around 34% of FDA‐approved drugs [133]. GPR91 was classified as an orphan receptor until He et al. identified succinate as a ligand for the receptor [134]. GPR91 is now known to be expressed in a variety of immune cell types, including monocytes and macrophages, adaptive immune cells (T cells and B cells) and dendritic cells (innate immune cells) [105, 135]. The role of GPR91 in the process of inflammation involves Gq and Gi proteins, which activate protein kinase c‐MAPK cascades and the inhibition of the cyclic adenosine monophosphate (cAMP) pathway that is responsible for the activation of PKA (Figure 4) [136, 137]. For example, GPR91 has been found to induce the metabolic reprogramming and pro‐inflammatory signaling pathways necessary for M1 macrophage polarization [135]. However, GPR91 has also been found to exert anti‐inflammatory effects in some cases. Attenuating GPR91 significantly reduces anti‐inflammatory actions in neuro stem cells and lowers pro‐inflammatory cytokine levels in white adipose [89, 138]. Several studies reported that GPR91 also induces M2 macrophage polarization [13, 138]. The discrepancy in succinate metabolism via GPR91 between studies could be partially explained by differences in macrophage subtypes and the surrounding environment, but, based on the results, how GPR91 regulates inflammation and macrophage polarization requires further study to develop a clear mechanistic map. In addition to that, the localization of succinate, whether it is intracellular or extracellular, may be another reason for its dual roles.

### 4.5 Succinylation Contributes to the Shift of Metabolic Pattern in M1 Macrophages

Succinylation has also been shown to play a key role in regulating several cellular processes, including energy metabolism, epigenetic coding, ion homeostasis, and many more [139, 140]. Succinylation reactions are posttranslational modifications (PTMs) that involve the addition of a succinyl group to lysine, one of the three amino acids with a positively charged side chain at physiological pH [139]. The reactions use succinyl‐CoA, which, as described above, is the product of the TCA cycle reaction catalyzed by KGDH. The succinate group is distinctly larger than the other PTM groups [102, 139], suggesting its crucial impact on the structure and function of the proteins it inhibits [139]. Succinylation was originally identified as a regulator of mitochondrial metabolism because the substrate, succinyl‐CoA, is generated by the TCA cycle [141, 142]. Succinylation regulates enzymes involved in the TCA cycle and the electron transport chain [143], where the modifications can ultimately alter glucose utilization and energy production. As mentioned in the previous section, a distinctive characteristic of M1 macrophages is a shift towards glycolysis and away from OxPhos; based on this, it is valid to further investigate the contribution of succinylation to M1 polarization. Finally, it is now known that the Succinylation of target proteins requires the sirtuin proteins (SIRT; e.g., SIRT5 in mitochondria), which couple succinyl moiety removal to NAD metabolism. The product of the SIRT reaction is a desuccinylated protein, nicotinamide, and succinyl‐O‐ADPR (O‐succinyl‐ADP‐ribose). ADP‐ribosyl hydrolase 3 (ARH3) then degrades succinyl‐O‐ADPR to ADP‐ribose and succinate.

## 5 KG Promotes M2 Traits, Opposing the Pro‐Inflammatory Effects of Succinate

**FIGURE 5:**
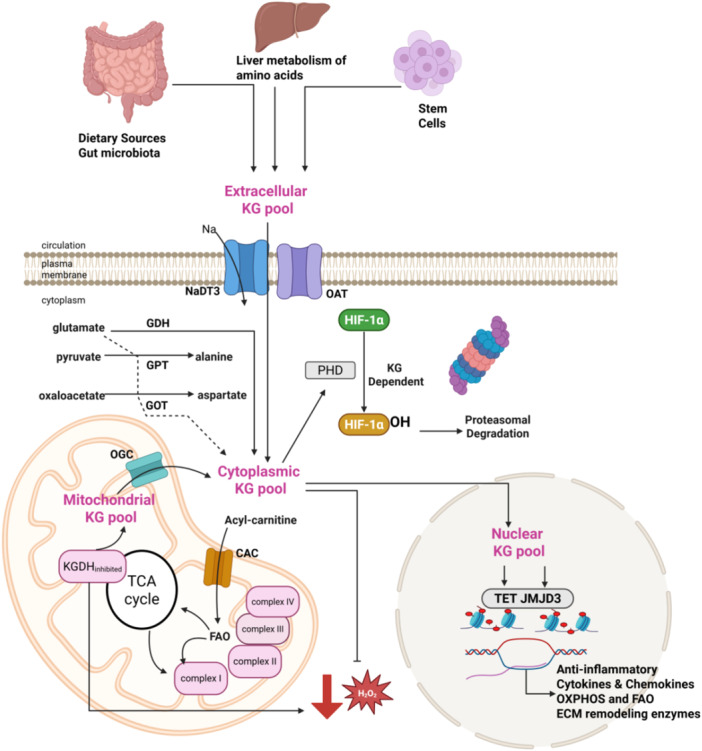
α‐ketoglutarate (KG) is a potent inducer of the anti‐inflammatory phase. The extracellular KG pool is highly dynamic and influenced by diet and gut microbiota, the efficiency of amino acid metabolism in the liver and the clearance of excess amino acids, and in certain cases stem cells. Sodium‐dicarboxylate cotransporter 3 (NaDC3) uses a sodium (Na) gradient to symport KG into the intracellular environment. Organic anion transporter (OAT) also imports KG. The cytoplasmic KG pool is also highly dynamic as it is influenced by amino acid metabolism and mitochondrial function. KG is generated by aminotransferases like glutamate‐oxaloacetate transferase (GOT) and glutamate‐pyruvate transferase (GPT), which use the amide in glutamate to generate alanine and aspartate. Glutamate dehydrogenase (GDH) forms KG from the oxidative deamination of glutamate. Mitochondria produce KG through the inhibition of KGDH activity, which results in its export to the cytoplasm by oxoglutarate carrier (OGC). Cytoplasmic KG has a multitude of effects. First, it can quench H_2_O_2_ directly through a spontaneous decarboxylation reaction. Second, it is a cofactor for prolyl‐hydroxylase, TET, and Jmjd3, as well as other KG‐dependent dioxygenases. This promotes HIF‐1α degradation and DNA and histone demethylations for epigenetic regulation, which triggers M2 polarization. The inhibition of KGDH also mitigates the buildup of H_2_O_2_, which prevents the induction of pro‐inflammatory signaling pathways.

Inhibition of KGDH promotes the M2 phase by limiting the production of succinate, succinyl‐CoA, and mtH_2_O_2_ (Figures 3 and 5). Limiting succinate production will mitigate PHD inhibition and GPR91 signal induction and prevent deactivation of JMJD3 and TETs (Figures 3 and 5). Abrogating H_2_O_2_ production would also prevent the sulfenylation of phosphatases, which in turn will result in the deactivation of NF‐κB, STAT1, STAT5, and ERK signals (Figures 3 and 5). On the other hand, inhibition of KGDH results in KG accumulation, which has also been shown to exert anti‐inflammatory effects in several studies [144, 145, 146]. First, KG is an antioxidant because the α‐keto acid group in the molecule can neutralize H_2_O_2_ in a spontaneous decarboxylation reaction (Figure 5) [147, 148]. KG is two enzymatic steps away from succinate in the TCA cycle but has immunoregulatory effects opposite to those of succinate. Indeed, in contrast to succinate, KG promotes M2 polarization and alleviates inflammation by preventing M1 cell differentiation or inducing M2 traits in M1 cells. This is because KG acts as a cofactor in the demethylation of histones and cytosine, thereby inducing the M2 phase through epigenetic reprogramming. KG could also limit inflammation through its interactions with GPR99. A recent study showed that succinylation of the gene encoding the pyruvate dehydrogenase E1 subunit leads to accumulation of KG in the tumor microenvironment, which activates the 2‐oxoglutarate receptor 1 (GPR99) on macrophages and leads to immunosuppression [149]. Finally, as described above, KG is an antioxidant that quenches H_2_O_2_ through a spontaneous decarboxylation reaction [150]. Therefore, in this section, we will focus on the anti‐inflammatory effects of KG and its ability to modulate macrophage polarization.

### 5.1 KG Is Required for 2OGDD Activity

KG is a substrate for and product of various metabolic pathways throughout the cell and therefore connects the TCA cycle to various other metabolic circuits (Figure 5). KG in macrophages is derived from the endogenous metabolism of various fuels (e.g., glucose, amino acids, fatty acids) or can be imported from the extracellular KG pool, which is dynamically influenced by several physiological and pathological factors (e.g., diet, gut microbiota, protein metabolism) (Figure 5). KG is an immunometabolite with anti‐inflammatory properties that, as described above, regulates FAO‐dependent oxygen consumption [149]. KG is a short‐chain carboxylic acid molecule and an important intermediate in the TCA cycle, responsible for energy metabolism, branched chain amino acid metabolism (BCAA), epigenetic regulation and oxygen sensing [146, 151, 152, 153]. KG is produced by the oxidative decarboxylation of isocitrate, a reaction catalyzed by isocitrate dehydrogenase (IDH) [145]. It is also produced via glutaminolysis, a two‐step reaction catalyzed by glutaminase and glutamate dehydrogenase, and through various transamination reactions, in which glutamate serves as a nitrogen donor for the biosynthesis of other amino acids [154]. The diverse role of KG, including its participation in anti‐inflammation, BCAA, and energy metabolism, can be explained by its molecular structure: it is a 5‐carbon oxo dicarboxylic acid with a reactive ketone at position 2. Glutamate, the primary amino acid in both animal and plant life, shares an almost identical structure with KG, except that it has an amine group rather than a ketone group on its second carbon. This explains why KG can be produced from glutamine or converted back to glutamate and eventually glutamine through glutamate dehydrogenase and glutamine synthetase [154]. Moreover, the carboxyl group of KG allows it to be decarboxylated with the assistance of enzymes, including KGDH and 2OGDD, a large group of enzymes that catalyze demethylation reactions or the hydroxylation of amino acids in proteins or metabolites [153]. 2OGDD catalyzes a diverse range of oxidative reactions, including collagen biosynthesis, transcriptional regulation, nucleic acid modification/repair, and secondary metabolite biosynthesis [121]. Many of the anti‐inflammatory and M2 traits promoted by KG are associated with distinct 2OGDDs located in the cytoplasm and nucleus of macrophages, as discussed in detail below.

### 5.2 KG Dampens Pro‐Inflammatory Pathways by Promoting HIF‐1α Degradation

KG is a necessary cofactor for PHD activity and is therefore the counterbalance to succinate‐assisted HIF‐1α stabilization (Figure 5) [155, 156]. KG supplementation has been shown to decrease HIF‐1α protein abundance by restoring normal PHD activity [157]. This effect was confirmed in two other studies where it was shown that KG prevents acute lung injury and liver injury by mitigating HIF‐1α activation [156, 158]. Together, this demonstrates that PHD activity, a member of the 2OGDD family of enzymes, depends on KG for the degradation of HIF‐1α. Notably, as mentioned in Section 5, the KG‐dependent hydroxylation of HIF‐1α by PHD, which also needs ascorbate and Fe to activate molecular oxygen for the hydroxylation reaction, produces succinate as a product of the reaction (Figure 5). It has been reported that KG prevents HIF‐1α stabilization in vivo by acting as an antioxidant. PHD enzymes are also inhibited by H_2_O_2_, and a recent study showed that stabilization of HIF‐1α in cells is prevented by KG‐mediated H_2_O_2_ neutralization, which correlates with decreased oxidative distress in cells [159]. HIF‐1α stabilization also occurs with loss of SDH activity, as in paragangliomas, pheochromocytomas, and solid tumors, indicating that succinate is a hypoxic signal. These results again suggest a regulatory mechanism between succinate levels and KG, and how both contribute to the inflammatory pathway.

### 5.3 KG Exerts Anti‐Inflammatory Effects by Suppressing the NF‐κB Pathway

KG is involved in multiple signaling pathways that promote the M2 phase. Dietary supplementation with KG inhibits the LPS‐induced NF‐κB‐mediated inflammatory pathway (Figure 5) [160]. Administration of dimethyl KG suppressed the RANKL‐activated NF‐κB pathway [161]. KG supplementation could abrogate pro‐inflammatory signals by spontaneously neutralizing H_2_O_2_, but, as described above and below, it could also be achieved by reactivating PHD or by inducing KG‐dependent demethylases that modulate epigenetic coding. However, we must point out that one study showed that glutamate dehydrogenase 1‐mediated KG production activates the NF‐κB signaling pathway under low‐glucose conditions, which is used to rewire cancer cell metabolism for tumorigenesis. KG also suppresses the mTORC1/P70S6K pathway to inhibit LPS‐induced M1 polarization [162]. Indeed, mTORC1 and extracellular signal‐regulated kinase (ERK)/nuclear factor erythroid factor 2‐related factor 2 (Nrf2) are also induced by KG, thereby mitigating oxidative distress, inducing mitochondrial biogenesis, and augmenting the expression of antioxidant genes [163, 164]. In conclusion, based on the targeted signaling pathway, KG plays crucial anti‐inflammatory and antioxidant roles in the physiological environment, which are opposite to those of succinate.

### 5.4 KG Regulates TET in Macrophages

As described above, the TET enzymes are KG‐dependent dioxygenases that catalyze the conversion of 5mC to 5ohC [165]. This means that KGDH activity and TCA cycle flux also orchestrate epigenetic regulation. In Section 4 we discussed that succinate inhibits TET enzyme activity as a product and antagonist generated from the binding of KG; therefore, KG/succinate plays a crucial role in regulating epigenetic coding by modulating TET activity (Figure 5). This also means the TET isozymes are key for regulating macrophage polarization (Figure 5). TET2 deficiency in the murine model leads to a 2‐50‐fold increase in M1‐associated markers, while the M2 gene remains mostly unchanged [166, 167]. However, it has been reported that TET2 downregulation promotes M2 polarization in an allergic rhinitis mouse model [168]. Although this finding does not directly reflect the inhibitory role of KG on M2 polarization, further investigation is needed into TET regulatory mechanisms and their effects on macrophage polarization. Like other dioxygenases, TET activity also needs ascorbate and Fe, which means H_2_O_2_ can disrupt the TET‐mediated demethylation of 5mC. This also represents a mechanism by which H_2_O_2_ promotes M1 polarization (e.g., by inhibiting TET and thereby regulating epigenetic programming to favor the M1 phase). As mentioned, KG can mitigate oxidative distress and improve mitochondrial function. Supplementing KG to human chondrocytes treated with pro‐inflammatory factor IL‐1 beta showed a significant decrease in H_2_O_2_ level observed using a fluorescence microscope [144]. However, KG can also regulate H_2_O_2_ production to induce ferroptosis in double‐hit lymphoma [169]. Combined with the fact that KG in cancer cell lines activates NF‐кB, these results suggested that KG's effect on H_2_O_2_ levels varies depending on different cell types and conditions. Indeed, KG is the upstream metabolite of succinate, a metabolite that can promote H_2_O_2_ production through reverse electron transport, and higher KG levels will increase succinate levels. Thus, it is important to not only consider KG but also the KG/succinate ratio when analyzing the signaling pathway and transcription regulation for cellular activities. KGDH, in this case, is a potent H_2_O_2_ producer in many cell lines, and the enzyme converting KG to succinyl‐CoA has been overlooked for years in its contribution to regulating macrophage polarization.

## 6 KGDH Activity Could be a Regulatory Nexus for Macrophage Polarization

So far, we have described how H_2_O_2_, KG, and succinate, as well as succinylation, which is mediated by succinyl‐CoA, are key factors regulating macrophage polarization. For example, the M1 phase is favored when the KG/succinate is low and H_2_O_2_ and succinyl‐CoA are abundant. By contrast, a high KG/succinate and suppression of H_2_O_2_ have the opposite effect, promoting M2 polarization. Notably, the availability of mtH_2_O_2_, succinate, KG, and succinyl‐CoA is controlled by KGDH. High KGDH activity produces mtH_2_O_2_, succinyl‐CoA and succinate, key immunometabolites produced from KG oxidation that drive M1 polarization. By contrast, suppressing KGDH activity would have the opposite effect by decreasing these pro‐inflammatory metabolites and increasing KG, which, as described above, is an anti‐inflammatory factor. This would suggest that KGDH is the regulatory nexus for controlling macrophage differentiation since its activity dictates the availability of these immunometabolites. KGDH is classically viewed as indispensable for energy metabolism and therefore only associated with TCA cycle flux [170]. However, growing evidence suggests it participates in many other cellular activities, including the propagation of intracellular signals. Figure 3 summarizes how we envisage KGDH serving as a regulatory nexus for macrophage polarization.

### 6.1 KGDH Is a Potent H_2_O_2_ Generator That Causes Oxidative Distress

Like other α‐keto acid dehydrogenase complexes, KGDH is composed of three subunits: the thiamine pyrophosphate‐dependent decarboxylase subunit (E1), the dihydrolipoamide acyltransferase (DLAT, E2), and dihydrolipoamide dehydrogenase (DLD, E3) [171]. The three subunits work together like three gears, using redox reactions to reduce NAD to NADH via the oxidative decarboxylation of the α‐keto acid. When converting the KG into succinyl‐CoA, the E1 subunit first binds to KG utilizing thiamine diphosphate (TPP) and catalyzes the oxidative decarboxylation of KG and the binding of succinic acid to the lipoic arm with sulfur residue on E2 [172]. E2 is the subunit that catalyzes the succinyl‐CoA production, which depends on the transfer of the succinyl moiety from TPP to the lipoic arm, followed by a disulfide exchange reaction with coenzyme A (CoASH) [173]. This produces dihydrolipoate in the E2 subunit. The E3 subunit is responsible for oxidizing the dihydrolipoate back to lipoate, liberating electrons that are then transferred to the NAD^+^ binding site of the E3 subunit through an FAD prosthetic group [173]. The E3 subunit is also the source of O_2_^•−^/H_2_O_2_ in KGDH. As mentioned, the ETC complexes were conventionally believed to be the major sources of H_2_O_2_. However, our team recently reported that inhibiting KGDH with 2‐keto‐3‐methylvaleric acid (KMV), an analog of KG, almost abolishes H_2_O_2_ production [85]. Valproic acid, another analog of KG, also strongly attenuates H_2_O_2_ production by KGDH [174]. Additionally, it was found that KGDH overproduces H_2_O_2_ in the liver mitochondria of mice with metabolic dysfunction‐associated steatotic liver disease induced by dietary fat overload [85]. Notably, the progression of MASLD is accompanied by increased inflammation driven by infiltrating monocytes and their differentiation into the M1 phenotype. Therefore, high KGDH activity in these invading macrophages could contribute to the advancement of MASLD to more serious forms of the disease. Consistent with the premise, KGDH is needed for macrophage activation. KMV addition to cultured microglial cells lowers H_2_O_2_ production and enhances cell viability under hypoxic conditions via inhibiting KGDH activity [175]. Moreover, S‐nitrosylation of KGDH was recently shown to promote M2 polarization [59]. Our team recently showed that KGDH S‐nitrosylation with mitochondria‐selective S‐nitrosylation agent, MitoSNO, strongly suppresses mtH_2_O_2_ production in isolated liver mitochondria and cultured Huh‐7 hepatocellular carcinoma cells [83]. The nitrosylation of the lipoic arm reduces mtH_2_O_2_ generation by blocking electron flow to the E3 subunit, further indicating that KGDH is a potential therapeutic target for mitigating liver disease [83]. This could serve as a means for the therapeutic prevention of inflammation since blocking KGDH with MitoSNO may prevent M1 polarization by decreasing mtH_2_O_2_ production [59, 83] (Figure 3). Other KGDH inhibitors like AA6 and siRNAs could also have similar therapeutic benefits, which was recently reviewed in [170]. Overall, KGDH may be important for macrophage polarization, and its inhibition with chemical antagonists or redox modifications could be used to treat inflammation.

### 6.2 KGDH Regulates the Epigenome and Cell Fate Decisions via Modifying the KG/succinate Ratio

As described above, succinate and KG are determinants for M1 and M2 polarization. KGDH uses KG as a substrate, producing succinyl‐CoA and then succinate, and therefore plays a prominent role in orchestrating the KG/succinate ratio. Therefore, it is highly likely that KGDH activity plays a crucial role in modulating macrophage cell fate decisions through the availability of mtH_2_O_2_, KG, and succinate. In a recent study that profiled macrophages harboring a heteroblastic mitochondrial tRNA^Ala^ mutation (*m.5019 A* > *G*), the mutation was found to decrease KGDH activity in bone marrow‐derived macrophages (BMDMs), corresponding to an increased KG/succinate [176]. Although the impact on BMDM polarization was not tested, it is feasible that the cells could favor an M2 phenotype (although the authors also found the mutation induces other cell alterations that may compromise macrophage functions). In a mouse model of Parkinson's disease, decreased KGDH protein levels are associated with increased KG levels [177]. As discussed before, both succinate and KG are involved in epigenetic modifications; thus, modulating their ratio makes KGDH likely to regulate cellular activity via these modifications. A recent article examining how metabolic adaptations direct cell fate reported that KGDH has a dual role, in which its activity is associated with bioenergetic and biosynthetic maintenance by regulating KG levels [178]. KGDH knockdown promoted the secretory lineage in an organoid enriched for intestinal stem cells, and a similar effect was observed with the addition of cell‐permeable dimethyl KG [178]. Like macrophages, intestinal stem cells can be differentiated into the lineages that make up the intestinal epithelium. It is fair to deduce that KGDH can work in a similar manner in the differentiation and polarization activities of macrophages as well by regulating its activity and modifying the KG level. Indeed, when comparing the KG and succinate levels between M1 and M2 macrophages, IL‐4‐stimulated macrophages, which possess an M2 phenotype, resulted in a higher KG/succinate ratio than the LPS‐induced M1 macrophages [12]. The mRNA results for these two groups also showed lower KGDH activity in the M2 group, suggesting that M2 favors KG accumulation via suppression of KGDH activity. Another crucial piece of evidence supporting KGDH's regulatory role is its presence in the nucleus [179]. KGDH has been reported to enter the nucleus in both mammalian cells and plant cells [179, 180], occupying regions of DNA where histone and cytosine methylations occur. A loss in functions KGDH leads to the hypo‐methylation of histones and cytosine due to the accumulation of KG and the induction of 2OGDDs like TET and Jumonji C‐domain‐containing histone demethylases (JMJs) as described above [180, 181, 182, 183]. This could impact macrophage polarization by promoting the M2 phase rather than the differentiation of proinflammatory M1 cells. Another point is that KGDH activity will govern the availability of succinate signaling, HIF‐1α stabilization, and GPR91 signaling, thereby determining the outcome of macrophage differentiation. Increased KGDH activity will supply mtH_2_O_2_ and succinate, promoting HIF‐1α stabilization and GPR91 signaling, while the decreased KG will mitigate M2 polarization (Figure 3). By contrast, temporary inhibition of KGDH will have the opposite effect, augmenting KG to promote M2 polarization through 2OGDD activation and limiting mtH_2_O_2_ (Figure 3). Taken together, it is feasible that KGDH is needed to facilitate macrophage polarization to the M1 or M2 phase (Figure 3).

## 7 Conclusion and Perspectives

Macrophage polarization depends on many factors, but recent work has shown that the TCA cycle‐linked metabolites KG and succinate are important immunometabolites that play opposite roles in macrophage polarization. In addition, mtH_2_O_2_ is also crucial for macrophage differentiation, with high mtH_2_O_2_ promoting a pro‐inflammatory state and low mtH_2_O_2_ having the opposite effect. Many of the nuances associated with how mtH_2_O_2_ signals promote M1 polarization are still being untangled, but what is known is that it functions as a suppressor of phosphatases that desensitize pro‐inflammatory signaling cascades. Crucially, the source(s) of H_2_O_2_ that induce redox signal transmission are still being elucidated. Because our team has identified KGDH as a potent mtH_2_O_2_ generator, we are proposing that this TCA cycle enzyme plays a key role in the transmission of pro‐inflammatory redox signals in macrophages. Together with its role in determining the availability of KG, succinyl‐CoA, and succinate, which we explained above, we contend KGDH is positioned to be a potentially important regulator of pro‐ and anti‐inflammatory macrophage signaling pathways. More research is needed to elucidate this potential signaling role for KGDH. But based on the available evidence, it is possible that KGDH serves as a regulatory nexus for the modulation of macrophage polarization in response to various physiological stimuli.

## Author Contributions

Meijing Li and Ryan J. Mailloux both contributed equally to the conception and design, and the writing and editing of the manuscript.

## Data Availability

Data sharing not applicable to this article as no datasets were generated or analyzed during the current study.
